# Mast cell tumours and other skin neoplasia in Danish dogs - data from the Danish Veterinary Cancer Registry

**DOI:** 10.1186/1751-0147-52-6

**Published:** 2010-01-22

**Authors:** Louise B Brønden, Thomas Eriksen, Annemarie T Kristensen

**Affiliations:** 1 Department of Small Animal Clinical Sciences, Faculty of Life Sciences, University of Copenhagen, Dyrlægevej 16, 1870 Frederiksberg C, Denmark

## Abstract

**Background:**

The Danish Veterinary Cancer Registry (DVCR) was established in May 2005 to gather information about neoplasms in the Danish dog and cat populations. Practitioners from more than 60 clinics throughout Denmark have submitted data on these species. The objectives of the current study were, with a special focus on mast cell tumours (MCT) to investigate the occurrence, gender distribution, biological behaviour, locations, types, the diagnostic method used and treatment of skin neoplasms in dogs based on information reported to the DVCR.

**Methods:**

From May 15^th ^2005 through February 29^th ^2008, reports on a total of 1,768 canine cases of neoplasia in the skin, subcutis or adnexa were submitted.) Of these, 765 cases (43%) were confirmed by cytology or histopathology.

**Results:**

The majority of dogs had a benign neoplasm (66%) while 21% were cases of malignant neoplasia. The most commonly encountered malignant neoplasms were MCT and soft tissue sarcomas and for benign neoplasms, lipomas and histiocytomas were the most common. The location of the neoplasms were primarily in the cutis, subcutis or in the perianal region. The occurrence, gender distribution, biological behaviour and location of canine skin neoplasias in Denmark were similar to earlier reports, although some national variations occurred. A correlation between grade of MCT and the proportion of cases treated surgically was observed.

**Conclusions:**

Population based cancer registries like the DVCR are of importance in the collection of non-selected primary information about occurrence and distribution of neoplasms. The DVCR provides detailed information on cases of skin neoplasms in dogs and may serve as a platform for the study of sub-sets of neoplastic diseases (e.g. MCT) or subgroups of the canine population (e.g. a specific breed).

## Introduction

The skin is the most common anatomical location of neoplasms and holds between 9.5% and 51% of all tumours in dogs [1-5]. A wide range of tumour types can be found in the skin, subcutaneous tissue and adnexa. However in most studies, the majority of the neoplasms in the skin were diagnosed as benign, e.g. adenomas and lipomas.

Diagnosis of skin tumours usually includes evaluation of cells using cytology and in cases where a biopsy is taken, histopathology. Furthermore a search for metastases is warranted. This approach is aimed at grading and staging the neoplastic disease.

Treatment of skin neoplasms, in particular malignant tumours, can be challenging. The treatment of choice in most cases includes surgical excision but depend on the type of cancer, stage, grade, and location. In some cases radiation or chemotherapy may be used alone or as adjunctive therapy of malignant neoplasms. This is typically the case in chemosensitive neoplasms in tumours that readily metastasise to distant locations or if complete resection can not be achieved e.g. distally on limbs or in the head.

Mast cell tumours (MCT), the most common skin cancer, represents a particular diagnostic and surgical challenge due to the high degree of behavioural variability from seemingly benign to highly malignant [6-8]. Fine needle aspirations of MCT are usually rich in cells as these exfoliate easily and cytology is usually sufficient for a diagnosis of MCT. However for grading of the tumours, histopathology is necessary. Suggested prognostic factors in MCT include location, grade and stage. Prediction of outcome and treatment planning should be based on a panel of prognostic factors, rather than on a single prognostic factor since no such has proven superior to predict MCT prognosis alone [9,10]. Treatment of MCT may include surgery, radiation, chemotherapy or a combination hereof. Dobson and Scase [2] concluded in a recent review that grade I MCT require only local resection and grade II MCT may be successfully treated with a 2 cm margin and one fascial plane deep resection. Grade III MCT carries a poorer prognosis but treatment outcome may improve if adjunctive radiation or chemotherapy is administered.

The objectives of the current study were, with a special focus on MCT to investigate the occurrence, gender distribution, biological behaviour, locations, types, the diagnostic method used and treatment of skin neoplasms in dogs reported to the Danish Veterinary Cancer Registry (DVCR). Furthermore the possible correlation between the diagnostic modality and the treatment elected will be investigated.

## Materials and methods

Canine cases entered in the DVCR from May 15^th ^2005 through February 29^th ^2008 were included in this study. They were selected based on location of a tumour in the skin, subcutaneous tissue or adnexa. The DVCR is a database of cases of neoplasia in Danish dogs and cats. It is an incident registry where each neoplasm is regarded as a separate entity, and data are collected prospectively. In contrast to other veterinary and human cancer registries DVCR comprises both benign and malignant neoplasms, and neoplasms diagnosed using other diagnostic methods than histology, such as cytology, diagnostic imaging etc. (Table 1). The base for the DVCR has been reported recently.

**Table 1:** Most common benign and malignant types of neoplasms in different studies.

Registry	Australia [15] #, N = 1000	Australia [16] #, N = 1000	Greece [10] #, N = 174	Korea [17] #, N = 748
**Distribution benign/malignant**	B: 41.9% M: 58.1%	B: 48.2% M: 52.8%	B: 53.4% M: 46.6%	B: 69% M: 31%
**Top 3 benign** **Pct of all skin tumours** **(Pct of all benign tumours)**	Basal cell tumour 12.0% (20.7%) Perianal gl. adenomas 8.3% (19.8%) Histiocytoma 7.8% (18.6%)	Histiocytoma 14.0% (29.0%) Lipoma 6.0% (12.4%) Basal cell epithelioma 5.5% (11.4%)	Hepato gl. adenoma 9.8% (18.3%) Lipoma 5.7% (10.8%) Histiocytoma 5.7% (10.8%)	Epidermal cysts 12.7% (18.3%) Lipoma 11.4% (16.4%) Histiocytoma 7.5% (10.8%)
**Top 3 malignant** **Pct of total skin (pct of all malignant skin tumours)**	MCT 17.6% (30.3%) STS 8.5% (14.6%) Melanoma 6.8% (11.7%)	MCT 16.1% (31.1%) STS 14.4% (27.8%) SCC 6.9% (13.3%)	MCT 13.8% (29.6%) STS 10.3% (22.2%) Basal cell carcinoma 4.0% (8.6%)	MCT 8.8% (28.7%) STS 3.3% (10.9%) Apocrine adenocarcinoma 3.1% (10.0%)

**Registry**		**Norway** [1] **#, N = 7401**	**South Africa** [18] **#; N = 903**	**USA** [3] **#, N = 277**

**Distribution benign/malignant**		M: 22%	B: 54.4% M: 45.6%	M: 100%
**Top 3 benign** **Pct of all skin tumours** **(Pct of all benign tumours)**		Epidermal adenoma 22.7% (29.2%) Lipoma 13.6% (17.4%) Histiocytoma 13.3% (17.1%)	Basal cell tumour 18.8% (34.6%) Perineal gland adenoma 13.4% (24.6%) Sebaceous gland adenomas and cysts 6.1% (11.2%)	-
**Top 3 malignant** **Pct of total skin (pct of all malignant skin tumours)**		MCT 10.0 (45.4%) STS 4.3% (19.6%) Malignant epidermal tumours 3.4% (15.4%)	SCC 16.4% (35.9%) Melanoma 11.4% (25.0%) MCT 8.9% (19.4%)	MCT (27%) Adenocarcinoma (26%) Melanoma (22%)

MCT: Mast cell tumours, SCC: Squamous cell carcinoma, STS: Soft tissue sarcoma

Data were entered into an Excel spreadsheet and statistical tests performed in SAS (version 9.1, SAS Institute, Cary, NC, USA). Chi-square test was used to analyse proportions of cases having had surgery and histopathology or cytology performed. *P *< 0.05 was considered to be significant.

## Results

A total of 1,768 cases of neoplasia in dogs were entered into the DVCR during the study period. 765 of these (43%, confidence interval (CI): 41.0-45.6) were located in the skin, subcutis or adnexa and had a diagnosis established by cytology or histopathology. Another 21 cases without a cytologically or histopathologically confirmed diagnosis were reported to the DVCRbut were omitted from the study.

Of the 765 cases, 400 cases (52%, CI: 48.8-55.8) were males, including 91 neutered), and 363 reports (48%, CI: 43.9-51.0) were on females, including 103 neutered. In 2 cases, information on gender was provided.

The majority of reports concerned benign tumours (66%, CI: 62.8-69.5, 506), while 21% (n = 160, CI: 18.0-23.8) were reports on malignant neoplasms. In 99 cases (13%, CI: 10.6-15.3) biological behaviour was not provided. The location of the neoplasms were primarily in the cutis, subcutis or in the perianal region (Table 2).

**Table 2:** Location and behaviour of neoplasms. Data from the Danish Veterinary Cancer Registry.

	Malignant No (Pct of location)	Benign No (Pct of location)	Unknown No (Pct of location)	Total No (Pct of total)
Cutis	108 (23)	294 (64)	58 (13)	460 (60)
Subcutis	16 (10)	128 (81)	15 (9)	159 (21)
Perianal	15 (20)	49 (66)	10 (14)	74 (10)
Glands	5 (19)	8 (31)	13 (50)	26 (3)
Eyelid	1 (5)	17 (90)	1 (5)	19 (3)
Other	15 (56)	10 (37)	2 (7)	27 (2)

Total	160 (20)	506 (66)	99 (14)	765 (100)

The most commonly encountered malignant neoplasms were MCT and soft tissue sarcomas (STS) and for benign neoplasms lipomas and histiocytomas (Table 3).

**Table 3:** Most commonly encountered neoplasms including the diagnostic method utilised and percentages of cases operated

Diagnostic method	Histology	Cytology	Other	Total
**Type of neoplasia**	**Number/Pct surgery**	**Number/Pct surgery**	**Number/Pct surgery**	**Number/Pct surgery**

Lipoma	18/89	164/23	6/33	188/29
MCT	65/53	49/91	2/100	116/75
Histiocytoma	46/98	52/44	2/100	100/70
Adenoma, all	42/98	51/33	2/100	96/63
STS	27/89	8/38	-	35/77
Melanoma	19/95	2/50	-	21/90

**Total**	**354/91**	**411/36**	**21/67**	**786/62**

Number: Total number of cases; Pct surgery: percentage of cases operated

MCT: Mast cell tumours, STS: Soft tissue sarcoma

The majority of cases (62%, CI: 58.4-65.3) were treated surgically (Table 3). Surgery was performed in 61% (CI: 56.2-64.7) of the benign cases and in 70% (CI, 62.9-77.1) of the malignant cases. The diagnostic method used to verify the diagnosis as well as the proportion of cases where surgery was performed is shown in Table 3. Microscopic evaluation by cytology or histopathology wwas the most commonly used diagnostic tool (Table 3). Corticosteroids were administered in 27 cases (4%, CI: 2.2-4.8). Thirteen of these cases were diagnosed as MCT and 5 cases were histiocytomas. In 6 cases of MCT and 1 case of malignant melanoma, corticosteroids were given in combination with surgery.

Euthanasia was the final outcome in forty patients (5%, CI: 3.7-6.8) of which 30 had malignant neoplasms, 6 had benign neoplasms and 4 had tumours of unknown biological behaviour.

In cases where the diagnosis was confirmed by histopathology, surgical excision was performed in 97% (CI: 94.7-99.2) of the benign tumours and in 80% (CI: 72.7-88.1) of the malignant ones, whereas in cases where the diagnosis was made by cytology, surgical excision was only performed in 31% (CI: 25.4-36.2) and 52% (CI: 38.9-64.6) of the benign and malignant cases, respectively. The proportion of cases diagnosed by histopathology that had surgical treatment was significantly higher than the proportion of surgical cases diagnosed by cytology (*P *< 0.0001).

### Mast cell tumours

MCT were the most common malignant neoplasms of the skin. A total of 114 MCT were reported, and their grade was reported in 51 cases. The diagnostic tool was cytology in 49 cases and histopathology in 65 cases. Only in two cases another diagnostic tool was chosen. Of the graded cases, 17 were reported as grade I, 26 as grade II and 8 as grade III. MCT were located on the trunk including the inguinal area in 46 cases (40%, CI: 31.4-49.4), in the perineal-genital area in 5 cases (4%, CI: 0.6-8.1) and on the extremities in 23 cases (20%, CI: 12.8-27.5). Surgery was performed in 83 out of the 114 cases (73%, CI: 64.6-81.0) including 34 cases of MCT on the trunk (74%, CI: 61.2-86.6), in 18 cases on extremities (78%, CI: 61.4-95.1) and in 2 cases of perineal-genital sites (40%, CI: 0-83.0). Of the graded cases, grade I MCT were excised in 11 cases (64.7%, CI: 42.0-87.4, of grade I cases), grade II in 25 cases (96.2%, CI: 88.8-100, of grade II cases) and grade III in 2 cases (25%, CII 0-55.0, of grade III cases). The majority of the grade III cases had either corticosteroid therapy (5 cases) or were euthanised (2 cases). In 14 cases of MCT, corticosteroid treatment was given supplementary to surgery. Fifteen cases had regional or distant metastases; of these 10 cases had surgery (Table 4).

**Table 4:** Distribution of 114 cases of MCT

	Graded as	Location	Surgery	Suppl. corticosteroids	Euthanasia
Grade I	17		11		

Grade II	26		25		

Grade III	8		2	5	2

Trunk incl. inguinal		46	34		

Perineal-genital		5	2		

Extremities		23	18		

## Discussion

Histopathology was used in 46% of all cases of skin neoplasia. Biopsy and histopathology without following surgery was only performed in 9.9% of cases but almost all cases having surgery had specimens submitted for histopathology. This illustrates a common linkage between these two events. The linkage is most likely due to a usual procedure including submission of biopsies from suspected malignant tumors and histopathological examination of excised malignant tumours post-operatively.

Cytology was widely used for diagnosis of skin tumours (54%) and should be included in the registry data as this method is often the only diagnostic procedure performed. This was especially true for benign neoplasms.

The proportion of neoplasms located in the skin was similar to data of a Norwegian study [1]. In an American study including both benign and malignant neoplasms, the proportion of tumours located in the skin was much lower (14.8%) [4]. If only malignant neoplasms were considered, the proportion of the malignant skin neoplasms was 21% in the current study, again equal to figures of the Norwegian registry (21.7%) [1]. Comparative data from other countries were either lower or higher (9.5% (Italy) *vs*. 30.3% (USA)) [3,5].

The male to female ratio (M:F) in this study was 1.1 in regards to all neoplasms as well as malignant neoplasms alone. This gender distribution was similar to that found in the Norwegian as well as a Greek study (M:F 0.85 and 0.89, respectively) [1,10], but very different to an Italian study in which a majority of females was seen (M:F 2.58) [1,5].

In other veterinary studies the majority of the skin neoplasms were benign as in the current study [1,2,4,10].

In the Norwegian study [1], the most common malignant neoplasia types were MCT (45.4% of all malignancies reported), STS (19.7%) and epithelial tumours (15.4%) (Table 1). These figures correspond to those found in the current study. The most common benign neoplasms in the Norwegian study [1]were epithelial tumours (29.2% of all benign tumours reported), lipomas (17.4%) and histiocytomas (17.1%), figures being similar to the current study. In the Italian study melanoma constituted only 4.1% of the skin malignancies [5], much lower than in the current study. The discrepancies among countries may be due to differences in dog populations, breed composition as well as cultural differences in animal management as illustrated previously [1,11].

### Mast cell tumours

A recent review [9], Dobson and Scase discussed the diagnostic and therapeutic options of cutaneous MCT. Prognostic factors of significance included grading (cytology and histopathology), staging (regional and distant metastases), breed, tumour localisation and treatment (surgery, radiation and chemotherapy). Cytological examination after fine needle aspiration is useful in establishing the diagnosis but histopathology is needed for grading [9].

Prognostic factors such as grade, metastases and tumour location are registered in the DVCR and the registry may be used for collection of cases for studies; both of cases that were treated following the recommendation for MCT treatment and cases that were not. The latter might provide an insight into the prognosis of animals where owners rejected surgery or other treatment options.

Grade II MCT were most often as recommended surgically excised. It was however noted that surgery was less often performed for both grade I and III tumours. Grade III tumours have a very poor prognosis even with treatment, which might discourage owners from extensive surgery. This may explain the discrepancy in surgery performed in MCT cases of different grades.

The location of MCT as a prognostic factor has been discussed in the literature and neoplasms in the mucocutaneous junctions and inguinal region have been reported as more aggressive and connected to a higher risk of metastasis than MCT in other locations [9]. However, no single prognostic factor has proven superior in the prediction of MCT prognosis [12]. Surgery was performed equally often on MCT located on the trunk and on extremities even though the latter location often impairs the use of wide surgery margins. Despite signs of metastases, indicating stage III or IV of the disease, 10 out of 15 cases had surgery performed. Surgery may be used as a palliative measure in the treatment of MCT as metastases can cause significant discomfort in the patient.

The treatment of choice in grade I and grade II MCT is surgery as recommended by Dobson and Scase [9] among others. Surgery should be performed with 2 cm margins [6,8]. In the current study surgery was performed in 73% of the MCT cases in the DVCR suggesting that cases of MCT are treated according to the recommendations at the participating veterinary clinics.

Treatment recommendation for high grade MCT includes both surgery and adjunctive therapy in the form of radiation or chemotherapy. This is consistent with the fact that half of the grade III cases had corticosteroid treatment. Apart from corticosteroid treatment and 1 case of chemotherapy, adjunctive therapy was not pursued in the MCT treatments reported to the DVCR. This is expected as both therapies are relatively new modalities in canine cancer treatment in Denmark compared to other countries [13,14].

Other prognostic factors for MCT have been investigated e.g. Ki67 index, c-KIT mutations, mitotic index, and surgical margins. These parameters were not included in the DVCR records as they are not routinely considered in primary practice diagnostic work-up. The DVCR may be used to locate cases for case-control studies or for follow-up studies of cases treated following certain protocols.

The DVCR offers an insight into the actual situation from primary practice clinics and hospitals among Danish dogs encountering skin neoplasms. Using MCT as an example, an attempt was made to outline the diagnostic modalities used and the treatments chosen when dogs are diagnosed (with MCT) outside a clinical trial setting. This knowledge can help uncover the consequences of non-consensus treatment, which is generally based on historical or empiric data. The choice of treatment is based upon the owners' choice and depends on many factors such as financial, ethical, social and animal welfare considerations. The latter, including survival time estimates, is an important part of the owner decision making and while clinical trials and controlled studies can contribute with information of the likely consequences of specific treatment regiments the DVCR might over time be able to uncover the consequences of the alternatives.

## Conclusions

The occurrence, gender distribution, histological malignancy and location of skin neoplasias in Denmark were in agreement with the majority of earlier reports, although some unexplained national variations seem to occur. The most commonly occurring malignant cutaneous neoplasms were MCT and STS.

Population based cancer registries like the DVCR are of importance in order to collect non-selected primary information of occurrence and distribution of neolasms. The DVCR provides detailed information on cases of skin neoplasms in dogs and may serve as a platform for the study of sub-sets of neoplastic diseases (e.g. MCT) or subgroups of the canine population (e.g. a specific breed).

## Competing interests

The authors declare that they have no competing interests.

## Authors' contributions

LBB carried out data management and statistical analysis, participated in designing the study, evaluating results, researching background literature and drafting the manuscript. TE participated in designing the study, evaluating results, researching background literature and drafting the manuscript. ATK participated in the coordination and drafting of the manuscript. All authors read and approved the final manuscript.
